# 

**Published:** 1893-07-08

**Authors:** 


**OUR NEW OFFICES**

428, Strand, W.C., will henceforth be the Office of this Journal.

**Notices of Births, Deaths, and Marriages,** are inserted in this column at a charge of 2s. 6d. for an announcement not exceeding four lines.

### NOTICE TO CORRESPONDENTS.

All MS., Letters, Books for Review, and other matters intended for the Editor should be addressed The Editor, The Lodge, Porchester Square, London, W.

The Editor cannot undertake to return rejected M.S., even when accompanied by stamped directed envelope.

**Notice to Advertisers and Subscribers.**

All Advertisements, Orders for Copies of The Hospital, and Business Communications should be addressed to The Publisher, *not to the Editor*, The Hospital, 428, Strand, W.C.

The Cost of Subscription, post free, is as follows:—Per week, 2½d.; per quarter, 2s. 6d.; per half-year, 5s.; per annum, 8s. 6d. Foreign:—Per annum, 10s. 8d.; payable, in all cases, in advance.

**"Burdett's Hospital Annual," 1893.**

Fourth year of publication. 700 pp. Boards, gilt lettering. A most complete guide to British, Colonial, Indian, and American Hospitals, Dispensaries, Nursing and Convalescent Institutions, and Asylums. Price 5s. Published by The Scientific Press (Limited), 428, Strand, W.C.

**NOTICE.—The Index for Vol. XIII. (ending March 1893) is on sale at the Office of this Journal, price 2½d., post free.**

### SCRAPS AND GLEANINGS.

The average life of the London omnibus horses is from four and a half to five and a-half years.

The building fund of the London School of Medicine for Women has received £3,000 from the Pfeiffer Bequest.

It has recently been discovered that some ants possess the most perfect sound-producing organs yet discovered in insects.

The World's Fair has been infested with enormous quantities of English sparrows, which have driven away nearly all the other birds.

The Queen has forwarded a donation of £25 to the Royal Hospital for Diseases of the Chest, City Road, E.C. Princess May has also sent £5.

In China pearls are artificially produced by placing small metallic objects in the interior of the living shell-fish, where they are covered by a pearly coating.

A butcher at Oddington, near Chipping Norton, has been sent to prison for eight months for selling unsound meat which caused the death of a customer.

There are at present in the elementary schools of England 938 certificated teachers at salaries under £40 a year. 2,2[illegible]3 at less than £45, 4,026 at less than £50, and 19,084 at less than £75.

The bazaar at the Westminster Town Hall in aid of the Alexandra Hospital for Children with Hip Disease, Queen's Square, was opened on July 3rd by the Prince and Princess of Wales.

Merton Abbey, the old-fashioned house at Mitcham, celebrated as the last residence of Nelson, is in the market. An effort is to be made to preserve it for the establishment of a Nelson museum.

The Martion Asylum and Infirmary Board held their annual meeting on June 16th. The treasurer's financial statement shewed a surplus for the year of £755 18s. 8d., an improvement upon last year.

Raw material to the value of £100,000 is got from the great Barnis reef of Australia and is exported annually. A chief item in this produce is pearl-shell for buttons and mother-of-pearl articles.

Sufficient gold has been procured from the ancient mines of Leadhills, Lanarkshire, Scotland, to furnish a keeper ring which the Princess May has graciously agreed to accept. The design is taken from the arms of Scotland.

We congratulate Mr. W. H. Collingridge, of the *City Press* (printers of The Hospital), on being elected a trustee of the Printers' Pension, Almshouse, and Orphan Asylum Corporation, in which he has always taken a great interest.

According to Benedictus Figulus, a German writer of the beginning of the seventeenth century, the philosopher's stone is composed of two things, body and spirit—mercurial water and *corpus solis*—which being interpreted means gold.

The Local Government Board have consented to the erection by the Metropolitan Asylums Board of a permanent fever hospital at Tottenham, on the site of which the temporary hospital has been standing during the past year.

The new wing of the Hospital for Sick Children, Great Ormond Street, contains four wards, providing 67 additional cots, extra accommodation for out-patients, additional nurses' dormitories, servants' rooms, and other offices.

A London builder has been fined for removing gravel from the site of a house and filling up with old mortar and straw, paper, and other rubbish. The magistrate said that such conduct was likely to spread fever in the metropolis.

The London and North-Western Railway are introducing corridor trains on the Scotch expresses. The trains have dining saloons for both first and third class passengers. In the third-class saloon a dinner of four courses is to be provided for 2s. 6d.

The publication of the first volume of a series of annual reports from the various teaching institutions of the Edinburgh Medical School is looked for. The Royal Infirmary, the Royal Hospital for Sick Children, the Maternity Hospital, and the Morningside Asylum are included in the scheme.

The Royal Caledonian Asylum, Holloway, was founded in 1815 for supporting and educating the children of soldiers, sailors, and marines, natives of Scotland, who had died or been disabled in the service of their country. Since its establishment about 2,000 children have been educated and brought up in the asylum.

An English writer of the 17th century says of Paris that phlebotomy was so much practised there that if one's little finger ached they opened a vein, and to balance the blood on both sides they usually let blood from both arms. For a cold in the head 50 ounces of blood were parted with in less than a fortnight.

The annual meeting of the subscribers to the Yorkshire Hospital for Incurable Diseases was held at Harrogate last week. The financial statement, which was read, covered a period of fifteen months' working. The receipts had been £601 9s. 5d., the expenditure £633 10s. 2d., leaving a debit balance to begin the year 1893.

At a recent meeting of the Croydon County Council the proposed infectious hospital was discussed. The engineer estimated the cost of buildings and fittings at £22,600, and the annual outlay at a basis of 40 patients, and including £1,200 for repayment of principal and interest, was estimated at £4,569. The consideration of the report of the committee was posponed to July 31st.

As a result of special observation, it is stated that urban fogs have a deleterious influence on plants under glass by the serious loss of light and by the presence of poisonous substances in the air. With stove plants especially the roots are not affected, and continue to absorb water, while the water is not given off by the leaves. Thus the tissues become gorged with water in a sort of dropsy.

Pure water with so small a trace of mercuric chloride in it that it would take more water than there is on our planet to make up with a pinch of the salt a solution of equal weakness, is fatal to many plants. One part of copper in a thousand million parts of water may be fatal to some plants. Such water has its poisonous properties destroyed by putting in a little gum or mud or charcoal.

### PUBLISHERS' ANNOUNCEMENTS.

2/6 *Demy 8vo. Cloth extra, 130 pp. Illustrated.* 2/6

Post free. Post free.

**MENTAL NURSING,**

BY

**WILLIAM HARDING, M.B.Edin., M.R.C.P.Lond.,**

*Assistant Medical Officer, Female Department, Berrywood, Northampton.*

DESIGNED SPECIALLY FOR THE INSTRUCTION OF ATTENDANTS UPON THE INSANE, AND NURSES IN CHARGE OF MENTAL CASES.

Nurses wishing to qualify themselves for the charge of mental cases will derive much instruction from this handy, inexpensive volume, which is beautifully printed in clear type, illustrated, and strongly bound in cloth.

The want of such a book has been widely felt, and the publishers recommend MENTAL NURSING with confidence to the managers of all institutions for the insane and to nurses wishing to take up mental cases.

"Nothing could be better devised to serve as a text-book. The lectures are so simple and so instructive."—*Local Government Journal.*

"The book is written in a clear, easily understood style."—*Glasgow Medical Journal.*

"The most suitable and thorough course of lectures on asylum nursing that has hitherto been published."—*Caledonian Medical Journal.*

London: THE SCIENTIFIC PRESS (Limited), 428, Strand, W.C.

Fourth Thousand, Cloth, Price 1s. 6d. nett, post free.

**QUESTIONS AND ANSWERS ON NURSING.**

By DR. J. W. MARTIN.

**LEONARD'S MANUAL OF BANDAGING.**

With 130 Engravings, Price 3s. 6d.

**THE HYGIENE OF BEAUTY.**

By DR. MONIN, of Paris.

Translated by MRS. CARDIVELL.

Price 3s. 6d.

BAILLIERE, TINDALL, and COX, King William Street, Strand.

*Catalogues Post Free.*

Profusely Illustrated, with Frontispiece; 220 pp. Price 2s. 6d.

**How to Become a Nurse,** AND HOW TO SUCCEED. A Complete Guide to the Nursing Profession for those who wish to become Nurses, and a Useful Book of Reference for Nurses who have completed their Training, and wish to Obtain Employment. Compiled by HONNOR MORTEN, Author of "Sketches of Hospital Life," "The Nurse's Dictionary," &c.

London: THE SCIENTIFIC PRESS (Limited), 428, Strand, W.C.

Price 6d., Post Free. Demy 16mo. 2ND EDITION. 7th Thousand. Price 6d., Post Free.

**THE NURSES' CASE BOOK.**

**For Use in Hospital as well as Private Nursing.**

*Suitable Size for the Apron Pocket.*

THIS Book is intended for the use of Nurses, that they may keep a brief record of their cases for their own instruction, as well as the record they have to keep for the medical attendant. Each sheet is ruled to last a week, and two sheets should usually be allowed for a case, though in typhoid it may be necessary to leave three, and in cases of common fracture one may be enough. It contains TWENTY-SIX COMPLETE TABLES, each being ruled to last ONE WEEK, for keeping an exact record of a Patient's condition, including Notes on TEMPERATURE, PULSE, RESPIRATION, MOTIONS, AGE, SEX, NAME, ADDRESS, OCCUPATION, TREATMENT, HISTORY, NAME OF MEDICAL ATTENDANT, NUMBER OF CASE, and DATE OF ADMISSION, &c., &c.

Nurses should combine together and order parcels of over a dozen to profit by the special discount terms—*i.e.*, 4s. 6d. PER DOZEN (POST FREE).

London: THE SCIENTIFIC PRESS (Limited), 428, Strand, W.C.

### PUBLISHERS' ANNOUNCEMENTS.

CHARLES GRIFFIN & Co.'s TEXT-BOOKS.
Now Ready. Handsome Cloth, 7s. 6d.
**THE DISEASES OF WOMEN** (OUTLINES OF).—A Text-book for Students. By JOHN PHILLIPS, M.A., M.D., F.R.C.P., Assistant Obstetric Physician, King's College Hospital; Physician, British Lying-In Hospital; Examiner in Midwifery, University of Glasgow, &c. With numerous illustrations.
*** In Dr. PHILLIPS' "Outlines," nothing is taken for granted. The Anatomy and Physiology of the subject are given clearly and simply, as forming the basis of Treatment, and the work throughout is so ESSENTIALLY PRACTICAL as to render it exceedingly valuable to all who have the care of gynæcological cases.

GRIFFIN'S POCKET BOOKS.

FIFTH EDITION. Pocket Size. Leather, 8s. 6d.

**A SURGICAL HANDBOOK.** By F. M. CAIRD, F.R.C.S., and C. W. CATHCART, F.R.C.S., Assistant Surgeons, Royal Infirmary, Edinburgh. With Illustrations.

"ADMIRABLY ARRANGED. The best practical little work we have seen."—*Edin. Med. Journal.*

Pocket Size. Leather, 8s. 6d.
**A MEDICAL HANDBOOK.** By R. S. AITCHISON, M.B.Edin., F.R.S.E. With numerous illustrations.
GENERAL CONTENTS: Case-taking—Diseases of the Circulatory System—Of the Respiratory System—Of the Renal System—Of the Digestive System—Of the Nervous System—Of the Hæmopoietic System—Fevers and Miasmatic Diseases—Constitutional Diseases—General Information and Useful Tables—Rules for Prescribing—Prescriptions.

SECOND EDITION. 6s.
**PRACTICAL SANITATION.** By GEO. REID, M.D., D.P.H., Medical Officer Staffs. County Council.
GENERAL CONTENTS: Water Supply and Pollution—Ventilation and Warming—Drainage, Sewage, and Refuse Removal—Sanitary and Insanitary Work and Appliances—Plumbers' Work—House Construction—Infection and Disinfection—Food.
"A VERY USEFUL handbook."—*San. Record.*
London: CHARLES GRIFFIN and Co., Limited, Exeter Street, Strand.

Price 6/- Post Free. *Demy 8vo. Nearly 200 pp. Cloth, Gilt.* Price 6/- Post Free.

*Profusely Illustrated with over 70 Special Cuts.*

THE
**ART OF MASSAGE.**
BY
**A. CREIGHTON HALE.**

THIS Book contains a complete exposition of the Art of Massage, together with many useful hints and much valuable information which every Masseuse should read. It comprises, in addition to the ordinary course of instruction, several original movements introduced by Mrs. Creighton Hale. It also deals with the Anatomy of the Human Body, clearly and concisely; the anatomical chapters being, like all the others, fully illustrated.

"The instructions and explanations given are admirably illustrated by a series of excellent original drawings . . . . these are so unusually good that they are almost sufficient of themselves to indicate what is to be done."—*The Westminster Review.*

"Appears to be all that is necessary for teaching the principles of the art . . . . A very full and clear statement of its subject."—*Queen.*

London: THE SCIENTIFIC PRESS (Limited), 428, Strand, W.C.

NOW READY.

2/6 Post Free. THIRD EDITION, *enlarged and partly re-written, cloth gilt, crown 8vo., about 200 pp.* 2/6 Post Free.

**HOSPITAL SISTERS AND THEIR DUTIES.**

BY

**EVA C. E. LÜCKES**

(*Matron of the LONDON HOSPITAL*).

THE favourable reception accorded to the first two editions of this useful and interesting work, and the regular demand for copies of it, encourage the publishers to place this third edition, greatly improved and enlarged, before the Institutions. The experience and position of its author entitle it to authority, and every Matron, Sister, or Nurse who aspires to become a Sister should read the advice and information contained in its pages.

Nearly 600 Copies of this Edition were sold within a week of publication.

The Press is unanimous in its Praise.

London: THE SCIENTIFIC PRESS (Limited), 428, Strand, W.C.

### FIRST IMPRESSIONS OF BOOKS RECEIVED.

[It is requested that all books intended to be noticed in this column may be sent to the Editor, at the Office, 428, Strand, W.C., not later than Tuesday evening in each week.]

The Medical Chronicle. June, 1893.

There is nothing very striking in the original articles this month. The Chronicle is well done, and records much of interest gathered from many sources. A useful journal.

Excision of Knee-Joint. By A. G. Miller, F.R.C.S.E., Surgeon to the Edinburgh Royal Infirmary.

This is a pamphlet containing articles previously published. It contains the lessons deduced from 30 cases of excision of the knee. It is well worthy of study, for the method of operating described seems to be a good one.

The Medical Pioneer. June, 1893.

This is the organ of the British Medical Temperance Association, and advocates the disuse of alcohol in health and disease. The editor succeeds in constantly imparting new interest to an old theme.

The Birmingham Medical Review. June, 1893.

Mr. Clay writes a practical, useful article on "Flat Foot—especially its Treatment." Mr. Gilbert Barling writes on what he calls "Appendicitis," a bastard word that is a disgrace to medical literature. It was imported into this country from abroad, but should be outlawed by every intelligent man. Mr. Barling does himself an injustice by using such a term, for his article is good.

Edinburgh Medical Journal. June, 1893.

A fair number. The opening article is by Dr. John Duncan on the "Surgical Treatment of Gall Stones." No new point is brought forward. There is a short paper, with illustrations, on three cases of Achondroplasia. Aphasia is discussed by Mr. John Wyllie, and there is a long article on "Allantoido-Angiopagous Twins," by Dr. Ballantyne—a rather tough morsel.

The Clinical Journal. June 7th.

This journal fully maintains its excellence. Every week it contains three or four good clinical lectures, and regular readers of it are kept well abreast of the time.

### Medical Vacancies and Appointments.

**APPOINTMENTS.**

J. D. Adams, M.D.St.And., M.R.C.S.Eng., appointed Medical Officer for the First (B) District of the Langport Union. G. T. Eccles, M.A., M.B., B.C.Camb., late House-Surgeon to the West London Hospital, appointed Medical Officer to the Workhouse of the Braintree Union. F. H. Edgeworth, M.B., B.B.Cantab., B.Sc.Lond., appointed Assistant Physician to the Bristol Royal Infirmary. W. H. Ellis, M.R.C.S., L.S.A., appointed Certifying Surgeon for the Shipley District. Mr. E. M. Goldie, appointed Assistant Medical Officer of the Poplar and Stepney Sick Asylum District. S. H. Habershon, M.A., M.D.Camb., appointed Assistant Physician to the Brompton Consumption Hospital. H. Hartley, L.R.C.P., L.R.C.S.Edin., appointed Medical Officer for the Norton District of the Malton Union. J. W. Harris, L.R.C.P.Lond., L.S.A., appointed Medical Officer for the Cranbrook and Frittenden Districts and the Workhouse of the Cranbrook Union. C. W. Hemming, L.R.C.P., L.R.C.S.Edin., appointed Medical Officer of Health for the Glyncorrwg Urban Sanitary District. Mr. H. Hodgson, appointed Medical Officer for the Blisworth District of the Towcester Union. J. S. Holden, M.D.Irel., L.R.C.S.Edin., reappointed Medical Officer of Health for the Borough of Sudbury. O. J. Kauffmann, M.D.Lond., appointed Medical Officer of the Workhouse Infirmary of the Parish of Birmingham. J. Kerr, M.A., M.D., D.P.H.Camb., appointed Medical Superintendent to the Bradford School Board. P. Kidd, M.A., M.D.Oxon, F.R.C.P.Lond., M.R.C.S.Eng., appointed Physician to the Brompton Consumption Hospital. W. Lake, D.P.H.Camb., M.R.C.S.Eng., appointed Medical Officer of Health for the Guildford Rural Sanitary Combined Districts. J. M. Martin, appointed Assistant Medical Officer of the Infirmary of the Greenwich Union. J. T. R. Miller, L.S.A., appointed *pro tem.* Medical Officer of Health to the Norton Urban Sanitary District of the Malton Union. C. A. Morton, F.R.C.S. Eng., appointed Demonstrator of Anatomy in the Bristol Medical School. Dr. W. A. Morton, appointed Medical Officer for the Shillington District of the Ampthill Union. C. Munden, M.R.C.S.Eng., L.S.A., appointed Medical Officer for the Third (C) District of the Langport Union. A. E. D. R. Peters, M.R.C.S. Eng., L.R.C.P.Lond., appointed Medical Officer of the Workhouse of the Midhurst Union. E. A. Pigott, L.R.C.P., L.R.C.S. Edin., L.S.A., appointed Medical Officer and Public Vaccinator for the Parish of Stoke-next-Clare, Suffolk. F. U. Purchas, M.D.Edin., reappointed Medical Officer for the Llanllwchaiarn District of the Newton Union. C. Randolph, L.R.C.P.Edin., M.R.C.S.Eng., appointed Medical Officer of Health for the Rural Sanitary District of the Wellington (Somerset) Union. S. W. Read. L.D.S., R.C.S., appointed Assistant Dental Surgeon to the National Dental Hospital. G. Renton, M.D.Edin., reappointed Medical Officer of Health for the Leadgate Urban Sanitary District. W. T. H. Spicer, M.A., M.B., F.R.C.S., appointed Ophthalmic Surgeon to the Metropolitan Hospital. F. Stephenson, L.F.P.S.Glasg., L.R.C.P.Edin., reappointed Medical Officer of Health to the Prestwich Urban Sanitary District. E. G. Stocker, L.R.C.P.Lond., M.R.C.S.Eng., appointed Medical Officer for the Third District of the Daventry Union. J. Tatham, M.D.St.And., F.R.C.P.Lond., appointed Consulting Physician to the Brompton Consumption Hospital. F. R. P. Taylor, M.B., B.S.Lond., M.R.C.S.Eng., L.R.C.P.Lond., appointed Third Assistant Medical Officer to the London County Asylum at Claybury. T. C. Temple, M.R.C.S.Eng., L.S.A., appointed Medical Officer for the Gravenhurst District of the Ampthill Union. W. A. Thomson, F.R.C.S., L.R.C.P.I., reappointed Medical Officer for the Hurley District of the Cookham Union. F. J. Waldo, M.D.Cantab., M.R.C.S.Eng., D.P.H.Eng., appointed Medical Officer of Health for the Middle Temple Sanitary District of the County of London. R. T. Williams, L.R.C.P., L.R.C.S.I., L.F.P.S.Glasg., appointed Medical Officer of Health for the Porthcawl Local Board. P. M. Yearsley, M.R.C.S.Eng., appointed Assistant Demonstrator in Anatomy to the Westminster Hospital Medical School. A. F. Voelcker has been elected an Assistant Physician to the Hospital for Sick Children in place of the late W. B. Hadden.

### CHARING CROSS HOSPITAL.

### MEDICAL VACANCIES AND APPOINTMENTS—(continued)

### VACANCIES.

The following vacancies are announced this week, the amount of salary in each case being given after the name of the institution:

**Resident Medical Officers.**

Central London Ophthalmic Hospital, 238A, Gray's Inn Road, N.W. House Surgeon. Rooms, coal, and light provided. Applications to the Secretary by July 10th.

Borough of Crewe. Medical Officer of Health; doubly qualified. Must reside in the borough, and devote his whole time to the duties of the office. Salary £200 per annum. Applications to Frederick Cooke, Town Clerk, Municipal Offices, Exchange Street, Crewe, by July 12th.

Essex and Colchester General Hospital, Colchester. House-Surgeon; doubly qualified; unmarried or widower without a family. Salary £100 per annum, with board and lodging. Applications to the Committee before noon on July 8th.

**Non-Resident Medical Officers.**

Caerphilly Local Board. Medical Officer of Health. Salary £40 per annum. Applications to David Lewis, Clerk to the Board, Caerphilly, by July 19th.

Charing Cross Hospital, W.C. Assistant Surgeon; must be F.R.C.S. Eng. Applications to the Chairman of Committee by July 24th.

Chelsea Hospital for Women, Fulham Road, S.W. Pathologist-Physician to Out-patients. Applications, on forms to be obtained at the Hospital, to the Secretary by July 8th.

Hospital for Epilepsy and Paralysis, 32, Portland Terrace, Regent's Park, N.W. Physician. Applications to the Secretary, H. Howgrave Graham, by July 15th.

Liverpool Dispensaries. Assistant Surgeon; unmarried. Salary £80 per annum, with apartments, board, and attendance. Applications to R. R. Greene, Secretary, by July 24th.

The Yorkshire College, Leeds. Demonstrator of Anatomy. Particulars from the Registrar.

### PASS LIST.

**University of Cambridge.**

EXAMINATIONS FOR MEDICAL AND SURGICAL DEGREES, EASTER TERM, 1893.

FIRST EXAMINATION.

PART I. CHEMISTRY AND PHYSICS.—Examined and approved: Ambrose, Cla.; Anderson, Trin.; H. L. Atkinson, B.A., Chr.; Bayly, Cla.; Bolland, Emm.; Boucher, Trin.; Bowen, Gonv. and Cai.; Bradley, Gonv. and Cai.; Brocke, B.A., Joh.; Browse, Cla.; F. E. Brunner, Gonv. and Cai.; H. N. Clarke, Gonv. and Cai.; J. S. Clarke, Gonv. and Cai.; Clarkson, B.A., H. Selw.; Darby, Trin.; Dobson, Pet.; Dore, Joh.; Edgell, King's; H. St. C. Elliott, Trin.; Fraser, Gonv. and Cai.; Garrood, Joh.; Green, Down; Grünbaum, Trin.; Gutch, B.A.; Chr.; Harmer, King's; Hartham, Jes.; Hedley, King's; Heilborn, Gonv. and Cai.; Helm, Magd.; A. C. Hill, Trin.; Hine, King's; Horne, B.A., Trin.; Houseman, Cla.; Kemp, Cla.; Leathes, B.A., Joh.; Le Cren, B.A., Chr.; Letchworth, Emm.; Mercer, Cla.; H. D. O'Sullivan, Emm.; J. A. O'Sullivan, Emm.; Pettinger, H. Selw.; Prest, Non Coll.; Pryce, Joh.; Reid, B.A., Joh.; Reissmann, Joh.; Rowland, H. Selw.; St. Leger, Gonv. and Cai.; Sanderson, B.A., Cla.; Scholberg, Trin.; Seowcroft, Gonv. and Cai.; Skrimshire, Joh.; Skyrme, Chr.; Slater, Trin.; Stabb, Down; Studd, Trin.; Sumner, Joh.; Sutcliffe, Emm.; Talbot, Trin.; W. H. A. Tebbs, Queens'; Turnbull, B.A., Joh.; Ware, Cai.; Walker, Queens'; White, Chr.; R. J. Willson, Emm.; E. L. Wilson, Cla.; Wingate-Saul, Trin.; Worlledge, B.A., Trin. H.

PART II. ELEMENTARY BIOLOGY.—Examined and approved: H. L. Atkinson, B.A., Chr.; A. P. Baker, Chr.; Barham, Gonv. and Cai.; Barnes, Chr.; Bateman, Down; Bemrose, Joh.; Borradaile, Gonv. and Cai.; Bowen, Gonv. and Cai.; Bradley, Gonv. and Cai.; F. E. Brunner, Gonv. and Cai.; H. N. Clarke, Gonv. and Cai.; Denyer, Queens'; Dobson, Pet.; Downer, Gonv. and Cai.; Edgell, King's; Ellis, Pet.; Fayrer, B.A., Trin.; Garrood, Joh.; Goode, Gonv. and Cai.; Griffith, Cla.; Grünbaum, Trin.; Harmer, King's; Harthan, Jes.; Harwood, Trin.; Hay, Gonv. and Cai.; Hedley, King's; Heilborn, Gonv. and Cai.; Helm, Magd.; A. C. Hill, Trin.; P. A. Hill, Gonv. and Cai.; Hine, King's; Kemp, Cla.; Knight, Emm.; Leathes, B.A., Joh.; Letchworth, Emm.; Lucas, Cla; Micklethwait, Trin.; Mills, Jes.; Nelson, B.A., Cla.; Orme, Gonv. and Cai.; H. D. O'Sullivan, Emm.; J. A. O'Sullivan, Emm.; Pettinger, H. Selw.; Plachecki, Chr.; Prest, Non Coll.; Prout, Down; Pryce, Joh.; J. F. Ralli, Gonv. and Cai.; Reissmann, Joh.; St. Leger, Gonv. and Cai.; Sanderson, B.A., Cla.; Sanderson, Jes.; Schreiner, Down; Sewell, Pemb.; Skyrme, Chr.; Slater, Trin.; H. M. H. Smith, Gonv. and Cai.; Sumner, Joh.; Sutcliffe, Emm.; Talbot, Trin.; Taylor, King's; B. N. Tebbs, Queens'; W. H. A. Tebbs, Queens'; Upward, Chr.; Walker, Queens'; Ware, Pemb.; Warren, Joh.; White, Chr.; Wilcock, Gonv. and Cai.; R. J. Willson, Emm.; Winton, Cla.; Worlledge, B.A., Trin. H.

Resident Medical Superintendent.

